# Probiotic Supplementation as an Alternative to Antibiotics in Bovine Sperm Cryopreservation: Effects on Motility, Oxidative Stress and Apoptosis

**DOI:** 10.1111/rda.70197

**Published:** 2026-03-19

**Authors:** Ali Doğan Ömür, Demet Çelebi, Serkan Ali Akarsu, Betül Apaydin Yildirim, Gamze Uçak, Tutku Can Acisu, Mehmet Akif Aydin, Sümeyye Başer, Özgür Çelebi, Recep Hakkı Koca, Gözde Arkali, Tarique Hussain

**Affiliations:** ^1^ Department of Reproduction and Artificial Insemination, Faculty of Veterinary Medicine Atatürk University Erzurum Turkey; ^2^ Department of Microbiology, Faculty of Veterinary Medicine Ataturk University Erzurum Turkey; ^3^ Department of Biochemistry, Faculty of Veterinary Medicine Atatürk University Erzurum Turkey; ^4^ Department of Reproduction and Artificial Insemination, Faculty of Veterinary Medicine Fırat University Elazığ Turkey; ^5^ Department of Reproduction and Artificial Insemination, Faculty of Veterinary Medicine Kafkas University Kars Turkey; ^6^ Department of Pharmaceutical Microbiology, Faculty of Pharmacy Erzincan Binali Yıldırım University Erzincan Turkey; ^7^ Department of Medical Microbiology, Faculty of Medicine Atatürk University Erzurum Turkey; ^8^ Department of Reproduction and Artificial Insemination, Faculty of Veterinary Medicine Bingöl University Bingöl Turkey; ^9^ Department of Physiology, Faculty of Veterinary Medicine Fırat University Elazığ Turkey; ^10^ Animal Sciences Division Nuclear Institute for Agriculture and Biology College, Pakistan Institute of Engineering and Applied Sciences (NIAB‐C, PIEAS) Faisalabad Pakistan

**Keywords:** alternative to antibiotics, bulls, cryobiology, probiotic strains, sperm quality

## Abstract

This study aimed to evaluate the effects of probiotic supplementation on bovine sperm cryopreservation by assessing post‐thaw spermatological and biochemical parameters. Twenty‐four ejaculations were collected from four Simmental bulls using the artificial vagina method. Ejaculates exhibiting over 70% motility were pooled to eliminate individual variations and allocated into five experimental groups: antibiotic‐free control group, antibiotic group, LA, BL and LR groups, respectively. Three probiotic strains, *Lactobacillus rhamnosus* (LR), *Lactobacillus acidophilus* (LA) and *Bifidobacterium longum* (BL), were incorporated into a Tris egg yolk‐based semen extender at a concentration of 10^9^ CFU/mL. Following dilution, samples were equilibrated and subsequently stored in liquid nitrogen. Post‐thaw evaluations were conducted for sperm motility and kinematic parameters using CASA, hypo‐osmotic swelling test, fluorescent staining for chromatin integrity, acrosomal integrity and mitochondrial membrane potential, as well as oxidative stress biomarkers. Additionally, apoptosis and metabolism related proteins were quantified by ELISA. The results exhibited that probiotic treated groups had significantly higher total and progressive motility as compared with controls (*p* < 0.001). In comparison with the control group, BL supplementation showed significantly reduced lipid peroxidation and increased antioxidant enzyme activities, along with lowered TGF‐β and Caspase‐3 levels, indicating attenuated oxidative stress and apoptotic signalling (*p* < 0.001). In contrast with the control group, LR supplementation resulted in elevated MDA and apoptosis‐related markers, suggesting strain specific pro‐oxidative effects (*p* < 0.001). There were no significant differences observed in STAR or ATP synthase levels across the treatments. Altogether, the results exhibited that strains of probiotics, particularly BL, have the potential to serve as an alternative to antibiotics in bovine semen cryopreservation. Nevertheless, strain‐dependent responses and limitations, especially with respect to DNA integrity, require further investigation.

## 1 Introduction

Bovine semen cryopreservation is a pivotal biotechnology that accelerates genetic improvement and reproductive efficiency in the global livestock industry (Khalil et al. 2018). However, the process inherently causes cryoinjury, necessitating the use of semen extenders to maintain sperm viability (Güngör et al. 2025). Historically, these extenders have been supplemented with a cocktail of antibiotics such as penicillin, streptomycin, and gentamicin to prevent microbial contamination (Schulze et al. 2018). However, their use is limited due to large levels of contamination (Stringfellow and Givens 2000) and the development of bacterial resistance to commonly used antimicrobials (Becher et al. 2013; Gloria et al. 2014; Visser et al. 1999). Antibiotics at different concentrations are reported to have negative effects on bull sperm (Gloria et al. 2014). Antibiotics used in semen extenders may reduce the number of other beneficial bacteria in the vaginal microbiota during artificial insemination, increasing the risk of colonisation by pathogenic microorganisms and negatively affecting the embryonic process (Lenický et al. 2022). Although antibiotics have long been used to control microbial contamination during sperm storage, safer alternatives are needed due to their cytotoxic effects and contribution to antimicrobial resistance (Morrell and Wallgren 2014).

Probiotics are microorganisms that have beneficial effects on host health by regulating the microbial balance of the intestinal tract (Çiftci and Tuna 2021). Probiotics are used to prevent the use of antibiotics and thus limit the emergence and spread of antibiotic‐resistant bacteria and antibiotic residues in meat and dairy products (Abu‐Tarboush et al. 1996). Commonly used probiotic microorganisms are *Bifidobacterium* spp. and *Lactobacillus* spp. species (Çiftci and Tuna 2021). *Lactobacillus* spp. are also a natural part of the vaginal microbiota (Borges et al. 2014). In addition, probiotic use in humans and animals improves sperm quality (Valcarce et al. 2017; Zhang et al. 2021). While the general protective potential of probiotics is acknowledged, it is crucial to recognise that the observed biological effects are highly strain‐specific. Different *Lactobacillus* and *Bifidobacterium* strains possess distinct metabolic pathways, cell wall components, and exopolysaccharide compositions, leading to differential modulations of the sperm microenvironment. Consequently, one strain may exert superior antioxidant effects by upregulating key enzymes, while another may primarily inhibit apoptosis or enhance membrane fluidity (Miao et al. 2024; Toscano et al. 2017).

Therefore, the present study aimed to evaluate and compare the effects of supplementing a Tris‐egg yolk extender with three distinct probiotic strains: *Lactobacillus rhamnosus* (LR), *Lactobacillus acidophilus* (LA), and *Bifidobacterium longum* (BL) at a standardised concentration (10^9^ CFU/mL). Specifically, we assessed their impact on post‐thaw sperm motility kinematics, membrane functionality, and molecular markers of oxidative stress (malondialdehyde (MDA) levels, superoxide dismutase (SOD) activity, and catalase (CAT) activity), apoptosis (Cysteine‐aspartic acid protease‐3 (caspase‐3)), mitochondrial bioenergetics (Adenosine triphosphate (ATP) synthase), inflammatory signalling (Transforming Growth Factor beta (TGF‐β)), calcium channel–associated regulation (Transient Receptor Potential Melastatin 3 (TRPM3)), and steroidogenic activity (Steroidogenic Acute Regulatory (STAR) protein), to identify the most suitable candidate for antibiotic‐free semen preservation.

## 2 Material and Methods

### 2.1 Ethical Approval

All procedures were approved by the Atatürk University Animal Experiments Local Ethics Committee (Protocol No: 2023/120).

### 2.2 Semen Collection

A total of twenty‐four ejaculations were obtained from four healthy Simmental bulls with reproductive capacity kept at the Atatürk University Food and Animal Husbandry Application and Research Center in Erzurum, Turkey, with six ejaculations obtained from per bull, ensuring an equal contribution from all subjects to the subsequent analyses. The bulls were maintained under uniform housing conditions and provided a total mixed ration supplemented with vitamins and minerals and provided water ad libitum. Before semen collection, the prepuce of each bull was cleaned and dried. The ejaculates were collected weekly for six consecutive weeks using a sterilised artificial vagina pre‐warmed to 42°C. The semen samples were evaluated macroscopically and microscopically and only samples exhibiting ≥ 70% total motility and ≥ 1 × 10^9^ sperm/mL were included (Bucak et al. 2015). These qualifying ejaculates were then pooled to eliminate individual bull variations and provide a homogenous semen batch. The pooled semen was subsequently divided into equal aliquots for dilution with the respective experimental extenders and cryopreservation.

### 2.3 Culturing of Probiotics

Probiotic cultures were prepared on MRS agar (Oxoid, UK). Plates containing 12 mL of MRS agar were inoculated with 2–3 mm loopfuls of probiotic extract. Lactobacillus spp. were incubated aerobically at 37°C for 24 h, whereas *Bifidobacterium* spp. was incubated anaerobically at 37°C for 72 h using the GasPak‐Anaerogen system (Oxoid Ltd., Basingstoke, UK). After incubation, colony morphology was examined and Gram‐stained smears were evaluated microscopically at 100× magnification (Olympus CBA, Melville, NY, USA). Lyophilised probiotic powders were stored at −20°C until use. For culture activation, 10 g/L skim milk powder (Molico, Nestlé, São Paulo, Brazil) was autoclaved at 121°C for 15 min; then 20 g/L glucose and 10 g/L yeast extract were added. Cultures were incubated at 37°C for 24 h before enumeration (Cheng et al. 2022; Hosseini et al. 2019; Toscano et al. 2017).

### 2.4 Bacteria Counting Procedures

One gram of lyophilised probiotic powder was added to 9 mL sterile saline (8.5 g/L NaCl) and serially diluted up to 10^−9^. Probiotic concentrations were standardised using a McFarland densitometer (Biomerieux DEN‐1B) immediately after dilution to achieve a final concentration of 1 × 10^9^ CFU/mL in the semen extender. Sequential post‐equilibration and post‐thaw CFU countings were not performed, as the study primarily focused on the functional response of spermatozoa to the initial standardised probiotic load during cryopreservation.

### 2.5 Experimental Groups

Ejaculates were divided into five experimental groups according to the composition of the semen extender.

#### 2.5.1 Antibiotic‐Free Control Group

Semen samples were diluted with a Tris–egg yolk extender without any antibiotics or probiotics [297.58 mM Tris (hydroxymethylaminomethane), 96.32 mM citric acid, 82.66 mM fructose, 15 mL egg yolk and distilled water to a final volume of 100 mL].

#### 2.5.2 Antibiotic Group

Semen samples were diluted with a Tris–egg yolk extender containing antibiotics [297.58 mM Tris (hydroxymethylaminomethane), 96.32 mM citric acid, 82.66 mM fructose, 15 mL egg yolk, 1,000,000 IU penicillin G potassium (I.E. Ulagay, İstanbul, Türkiye), 100 mg streptomycin sulfate (I.E. Ulagay, İstanbul, Türkiye) and distilled water to a final volume of 100 mL].

#### 2.5.3 LA Group

Semen samples were diluted with antibiotic‐free Tris–egg yolk extender supplemented with *Lactobacillus acidophilus* at a final concentration of 1 × 10^9^ CFU/mL.

#### 2.5.4 BL Group

Semen samples were diluted with antibiotic‐free Tris–egg yolk extender supplemented with *Bifidobacterium longum* at a final concentration of 1 × 10^9^ CFU/mL.

#### 2.5.5 LR Group

Semen samples were diluted with antibiotic‐free Tris–egg yolk extender supplemented with *Lactobacillus rhamnosus* at a final concentration of 1 × 10^9^ CFU/mL.

### 2.6 Bacterial Load Count in Sperm Diluents

Bacterial density was quantified via serial dilution and colony counting. The optimum probiotic dose was standardised as 10^9^ CFU/mL, based on prior reports (Cheng et al. 2022; Hosseini et al. 2019; Toscano et al. 2017). This concentration provided the probiotic‐induced antibacterial effect required for subsequent experiments.

Following dilution, semen samples were loaded into 0.25 mL plastic straws and maintained at 24°C for 10 min. The straws were then equilibrated at +5°C for 3 h, frozen for 10 min in nitrogen vapour (approximately −100°C, 4 cm above liquid nitrogen) and finally stored in liquid nitrogen at −196°C until further analysis.

### 2.7 CASA Analysis

Post‐thaw motility and kinematic parameters were evaluated using a CASA system (ISAS II, Proiser, Spain). Semen straws were thawed in a water bath at 37°C for 25 s. A 3 μL aliquot of thawed semen was placed on a pre‐warmed SpermTrack‐20 slide and examined. The specific software settings for bull spermatozoa were configured as follows: particle size area of 5 to 70 μm^2^, connectivity of 12 and a minimum of 10 images to calculate ALH. The velocity thresholds were classified as slow (10–25 μm/s), medium (25–50 μm/s) and rapid (> 50 μm/s). Spermatozoa with a straightness (STR) of 70% or higher were considered progressive. An average of 120 to 200 spermatozoa were analysed for total motility, progressive motility and kinematic parameters (Sönmez and Firat 2022).

### 2.8 Hypo‐Osmotic Swelling Test (HOST)

Plasma membrane integrity was examined using the hypo‐osmotic swelling test (HOST). Briefly, 30 μL of semen was incubated with 300 μL of a 100 mOsm hypo‐osmotic solution at 37°C for 60 min. After incubation, 30 μL of the mixture was assessed in a phase‐contrast microscope to analyse the percentage of spermatozoa exhibiting tail swelling (Büyükleblebici et al. 2014).

### 2.9 Mitochondrial Membrane Potential

Mitochondrial membrane potential was determined using the JC‐1 fluorescent probe. A total of 2.5 μL JC‐1 and 2.5 μL propidium iodide (PI) were added to 300 μL of thawed semen and incubated for 20 min at 37°C in the dark. After incubation, samples were fixed with 10 μL of Hancock's solution (10 mL formalin, 0.85 g NaCl, 0.4 g Tris and 89.15 mL distilled water). Slides were then examined in a fluorescence microscope (Zeiss Axioscope A1, Germany) to evaluate mitochondrial membrane potential (Öztürk et al. 2017).

### 2.10 Spermatozoon DNA Damage

Sperm DNA fragmentation was examined using the acridine orange (AO) assay. Briefly, 20 μL of semen was smeared onto glass slides and air‐dried. The slides were then fixed overnight in freshly prepared Carnoy's solution (methanol: acetic acid, 3:1), re‐dried and stained with acridine orange for 3 min. The stained slides were examined in a fluorescence microscope (Zeiss Axioscope A1, Germany). Approximately 120–150 spermatozoa were evaluated to determine DNA integrity (Nur et al. 2010).

### 2.11 Acrosomal Integrity

Acrosomal integrity was evaluated using fluorescein isothiocyanate–labelled peanut agglutinin (FITC‐PNA) staining. A stock solution was prepared by dissolving 120 μg FITC‐PNA in 1 mL phosphate‐buffered saline (PBS). A 60 μL aliquot of thawed semen was mixed with 10 μL FITC‐PNA and 2.5 μL propidium iodide (PI) and incubated at 37°C for 20 min in the dark. Then, after incubation, samples were fixed with Hancock's solution and examined under a fluorescence microscope. At least 120–150 spermatozoa were evaluated to assess acrosomal integrity (Bucak et al. 2015).

### 2.12 Oxidant and Antioxidant Analysis of Thawed Sperm

For biochemical evaluations, the entire content of the thawed semen straws, which were previously standardised to contain 20 × 10^6^ spermatozoa per straw, was directly utilised without a washing step. The whole semen‐extender mixtures were homogenised for 1 min using a Qiagen TissueLyser LT and subsequently centrifuged at 400 × g for 15 min (Ünal and Uysal 2024). Then the supernatants were used for the determination of malondialdehyde (MDA) levels (Yoshioka et al. 1979), superoxide dismutase (SOD) activity (Sun et al. 1988) and catalase (CAT) activity (Goth 1991). All measurements were performed using a BioTek ELISA reader.

### 2.13 ATP, TRPM3, STAR, TGF‐β and Caspase‐3 Analyses

The concentrations of Adenosine triphosphate (ATP), Transient Receptor Potential Melastatin 3 (TRPM3), Steroidogenic Acute Regulatory protein (STAR), Transforming Growth Factor beta (TGF‐β) and Cysteine‐aspartic acid protease‐3 (caspase‐3) were determined using commercially available Enzyme‐Linked Immunosorbent Assay (ELISA) kits according to the manufacturers' instructions. The kits used were Bovine Caspase‐3/CPP32 (SunRed Biological Technology Co. Ltd., China; Cat. No. 201‐04‐0257), Bovine STAR (SunRed; Cat. No. 201‐04‐3742), Bovine TRPM3 (SunRed; Cat. No. 201‐04‐3314), Bovine TGF‐β (SunRed; Cat. No. 201‐04‐0011) and Bovine ATP Synthase Mitochondrial F1 Complex Assembly Factor 2 (BT Lab; Cat. No. E0464Bo). Absorbance was measured at 450 nm using a BioTek ELISA reader.

### 2.14 Statistical Analysis

A prior power analysis was performed using G*Power 3.1 software to estimate the minimum sample size required to detect medium effect sizes (*f* = 0.25) with a statistical power of 0.80 and an alpha level of 0.05. The calculated sample size was consistent with the number of replicates used in the present study. All data were analysed using IBM SPSS Statistics (version 26.0; IBM Corp., Armonk, NY, USA). Before statistical analysis, the normality of data distribution and homogeneity of variances were evaluated using the Shapiro–Wilk and Levene's tests, respectively. Differences among experimental groups were evaluated by one‐way analysis of variance (ANOVA) followed by Tukey's post hoc multiple comparison test. Data are expressed as mean ± standard error of the mean (SEM) and statistical significance was accepted at *p* < 0.05.

## 3 Results

### 3.1 Sperm CASA Parameters

Table 1 shows the influence of probiotic treatments on CASA parameters of post‐thaw Simmental bulls. The results revealed that total and progressive motility were lower in control and antibiotic groups, whereas both parameters were significantly higher in all probiotic‐supplemented groups (*p* < 0.001). The proportion of rapid spermatozoa was also significantly greater in the probiotic‐treated groups as compared to the control and antibiotic groups (*p* < 0.001). In contrast, the control group without antibiotics exhibited a significantly lower percentage of spermatozoa moving at medium velocity compared with the antibiotic group and other probiotic‐supplemented groups (*p* < 0.001). The LR group significantly showed a higher proportion of slow‐moving spermatozoa relative to the antibiotic‐free control and other groups (*p* < 0.001). Interestingly, the percentage of static spermatozoa was significantly elevated in the control and antibiotic group compared with all probiotic groups (*p* < 0.001).

**TABLE 1:** Effect of probiotic treatments on post‐thaw sperm CASA parameters in Simmental bulls.

	Antibiotics‐free control group	Antibiotic group	LA group	BL group	LR group
Total motility (%)	42.81 ± 0.46^A^	49.03 ± 2.83^AB^	54.40 ± 4.17^bc^	59.68 ± 2.69^C^	58.16 ± 0.53^C^
Progressive motility (%)	31.83 ± 0.96^A^	31.85 ± 2.00^A^	39.31 ± 4.14^B^	41.21 ± 2.61^B^	40.46 ± 0.72^B^
Rapid (%)	38.03 ± 0.98^A^	37.96 ± 1.85^A^	45.43 ± 4.79A^B^	49.81 ± 2.53^B^	47.90 ± 1.08^B^
Medium (%)	2.48 ± 0.49^A^	7.41 ± 0.77^B^	5.40 ± 0.65^B^	6.40 ± 0.74^B^	5.83 ± 0.69^B^
Slow (%)	2.31 ± 0.34^A^	3.70 ± 0.58^AB^	3.56 ± 0.66^AB^	3.43 ± 0.54^AB^	4.43 ± 0.90^B^
Static (%)	57.20 ± 0.46^C^	50.96 ± 2.83^bc^	45.60 ± 4.17^AB^	40.31 ± 2.69^A^	41.83 ± 0.53^A^
VCL (mm/s)	120.30 ± 4.21^B^	101.38 ± 2.39^A^	111.00 ± 3.62^AB^	110.91 ± 3.58^AB^	111.81 ± 4.58^AB^
VSL (mm/s)	60.73 ± 1.05^B^	52.76 ± 1.27^A^	57.18 ± 1.81^AB^	56.55 ± 2.95^AB^	59.98 ± 1.98^B^
VAP (mm/s)	74.03 ± 1.90^B^	65.31 ± 1.27^A^	70.33 ± 1.83^AB^	70.56 ± 3.28^AB^	73.00 ± 2.35^B^
LIN (%)	50.78 ± 1.90	52.20 ± 1.65	51.78 ± 2.22	50.88 ± 1.55	53.75 ± 0.81
STR (%)	82.15 ± 1.39	80.81 ± 0.79	81.31 ± 1.32	80.03 ± 0.90	82.13 ± 0.38
WOB (%)	61.73 ± 1.58	64.51 ± 1.70	63.60 ± 1.97	63.46 ± 1.30	65.40 ± 0.81
ALH (mm)	4.71 ± 0.15	4.26 ± 0.14	4.48 ± 0.20	4.45 ± 0.10	4.41 ± 0.18
BCF (Hz)	11.06 ± 0.30	10.81 ± 0.24	11.20 ± 0.39	10.95 ± 0.35	10.96 ± 0.26

*Note:* A, B, C, D: Indicates the difference between groups. *p* < 0.001 values are given as mean ± standard error.

Abbreviations: ALH, Amplitude of lateral head displacement; BCF, Cross frequency crossover; BL, *Bifidobacterium longum* ; LA, *Lactobacillus acidophilus* ; LIN, Linearity; LR, *Lactobacillus rhamnosus* ; STR, Track direction; VAP, Mean path velocity; VCL, Curvilinear velocity; VSL, Straight‐line velocity; WOB, Skew.

The sperm velocity parameters such as curvilinear velocity (VCL), straight‐line velocity (VSL), and average path velocity (VAP) tended to be higher in probiotic‐treated groups in relation to antibiotic‐free control and antibiotic groups, with the highest values generally observed in the LR and LA groups. In contrast, the antibiotic group consistently showed the lowest velocity values. The sperm movement parameters such as linearity (LIN), straightness (STR), and wobble (WOB) were comparable among all groups, indicating that probiotic supplementation mainly enhanced sperm speed rather than altering movement trajectory. Similarly, amplitude of lateral head displacement (ALH) and beat cross frequency (BCF) had not markedly influenced among id treatments.

### 3.2 Sperm Functional Integrity Parameters

The impact of probiotic treatments on freeze–thawing sperm membrane functional integrity parameters of Simmental bulls is displayed in Table 2. DNA fragmentation was significantly highest in the BL and LA groups compared with the antibiotic free control group whereas the lowest values were significantly observed in the antibiotic and LR groups respectively (*p* < 0.001). The acrosome damage was significantly higher in LR, antibiotics, and BL groups while the lowest percentage values were noticed in the LA group and antibiotic free control group respectively (*p* < 0.001), suggesting the LA group was efficient in minimising acrosomal damage. The mitochondrial membrane potential significantly exhibited the same higher trend in LR, LA, and antibiotic groups whereas the highest trend was observed in the BL group as compared to the antibiotic free control group (*p* < 0.001). The plasma membrane functional integrity exhibited that the antibiotic, LA, and BL groups remained significantly the same while the higher group was significantly elevated in the LR group in response to the antibiotic free control group (*p* < 0.001).

**TABLE 2:** Impact of probiotics on freeze‐thaw sperm functional integrity parameters in Simmental bulls.

	Antibiotics‐free control group	Antibiotic group	LA group	BL group	LR group
DNA fragmentation	50.25 ± 1.37^B^	41.50 ± 3.57^A^	78.00 ± 1.58^C^	87.00 ± 2.48^D^	42.00 ± 2.16^A^
Acrosome damage	40.25 ± 2.39^A^	69.75 ± 7.48^B^	29.25 ± 3.01^A^	63.75 ± 4.71^B^	75.50 ± 6.33^B^
Mitochondrial membrane potential	32.25 ± 4.32^A^	49.75 ± 9.88^AB^	59.25 ± 10.77^AB^	78.50 ± 11.76^B^	59.50 ± 11.01^AB^
HOST test	20.50 ± 3.12^A^	24.00 ± 3.24^AB^	30.25 ± 3.47^AB^	26.25 ± 3.14^AB^	32.00 ± 3.34^B^

*Note:* A, B, C, D: Shows the difference between groups. *p* < 0.001 values are given as mean ± standard error.

Abbreviations: BL, *Bifidobacterium longum* ; LA, *Lactobacillus acidophilus* ; LR, *Lactobacillus rhamnosus* .

### 3.3 Biochemical Analysis

The results of oxidant and antioxidant parameters of probiotic supplemented groups in freeze–thawing Simmental bulls are illustrated in Table 3. The significantly lowest MDA level was observed in the BL group, whereas the highest value was recorded in the LR group (*p* < 0.001). The SOD and CAT activities were significantly higher in the BL, LA, and antibiotic groups as compared to the antibiotic‐free control and LR groups (*p* < 0.001).

**TABLE 3:** Influence of probiotics on post‐thaw antioxidant and oxidant parameters in Simmental bulls.

	Antibiotics‐free control group	Antibiotic group	LA group	BL group	LR group
MDA (mmol/L)	32.66 ± 2.25^C^	24.58 ± 1.37^AB^	28.35 ± 2.10^bc^	22.83 ± 0.63^A^	47.91 ± 1.17^D^
SOD (U/mL)	21.45 ± 1.26^A^	45.53 ± 1.12^C^	36.30 ± 1.30^B^	49.04 ± 1.80^C^	18.30 ± 0.60^A^
CAT (KU/L)	120.08 ± 2.53^A^	226.68 ± 7.77^B^	219.96 ± 3.16^B^	244.22 ± 5.86^C^	115.06 ± 0.54^A^

*Note:* A, B, C, D: Shows the difference between groups. *p* < 0.001 values are given as mean ± standard error.

Abbreviations: BL, *Bifidobacterium longum* ; LA, *Lactobacillus acidophilus* ; LR, *Lactobacillus rhamnosus* .

The results of probiotic supplemented groups in post‐thaw semen biochemical parameters for ATP, TRPM3, STAR, TGF‐β, and Caspase‐3 are presented in Table 4. TGF‐β concentrations were lowest in the BL group and highest in the LR group (*p* < 0.001). TRPM3 levels were significantly higher in the BL, LA, and antibiotic‐free control groups compared with the LR group (*p* < 0.001). Similarly, Caspase‐3 levels were lowest in the BL and antibiotic groups, whereas the highest level was found in the LR group (*p* < 0.001). The STAR and ATP synthase were detected as non‐significant across the experimental groups (*p* > 0.05).

**TABLE 4:** Effect of probiotics treatments on ATP synthase, TRPM3, STAR, TGF‐β, and Caspase‐3 parameters in Simmental bulls.

Parameters	Antibiotics free control group	Antibiotic group	LA group	BL group	LR group
TGF‐β (ng/mL)	9.04 ± 0.22^bc^	8.39 ± 0.16^AB^	8.80 ± 0.44^B^	7.70 ± 0.35^A^	10.00 ± 0.44^C^
TRPM3 (ng/mL)	75.28 ± 1.27^AB^	80.92 ± 4.39^B^	78.03 ± 0.99^AB^	80.90 ± 2.81^B^	71.39 ± 1.05^A^
STAR (pg/mL)	184.30 ± 6.02	194.89 ± 2.08	210.38 ± 16.12	210.98 ± 15.42	182.45 ± 5.14
Caspase‐3/CPP32 (ng/mL)	1.03 ± 0.03^AB^	0.90 ± 0.03^A^	1.05 ± 0.00^AB^	0.84 ± 0.03^A^	1.17 ± 0.16^B^
ATP‐synthase (ng/mL)	2.38 ± 0.19	2.48 ± 0.40	2.37 ± 0.41	2.90 ± 0.49	1.81 ± 0.15

*Note:* A, B, C: Shows the difference between groups. *p* < 0.001 values are given as mean ± standard error.

Abbreviations: BL, *Bifidobacterium longum* ; LA, *Lactobacillus acidophilus* ; LR, *Lactobacillus rhamnosus* .

## 4 Discussion

Cryopreservation inevitably compromises sperm quality after the freezing–thawing process in cattle, as observed in other species (Khalil et al. 2018). The major contributing factor is the extreme physicochemical stress encountered during cooling and ice crystal formation, which alters the sperm membrane environment (Khalil et al. 2018). To minimise microbial contamination, various antibiotics are commonly incorporated into semen extenders (Dissanayake et al. 2014). However, accumulating evidence indicates that such antibiotics may exert cytotoxic effects on spermatozoa and contribute to antimicrobial resistance (Seneca and Ides 1953). Probiotics, particularly *Lactobacillus* spp. and *Bifidobacterium* spp., are beneficial microorganisms of the normal microbiota known to suppress pathogenic bacteria such as *Escherichia coli* (Blum et al. 2002). The present study therefore investigated the effects of LR, LA and BL at a concentration of 10^9^ CFU/mL on the cryopreservation outcomes of bull sperm.

Sperm motility is considered a critical indicator of sperm fertilising potential (İnanç et al. 2022). However, the effect of antibiotic supplementation on motility remains contradictory: while some previous research suggests it can reduce motility in buffalo and bull semen (Hasan et al. 2001), another study has reported no detrimental effects (Gloria et al. 2014). Furthermore, the use of antibiotics during semen processing may foster antimicrobial resistance (Goularte et al. 2020). Natural substitutes such as honey have been shown to improve motility and preserve viability compared with conventional antibiotics (Nasreen et al. 2020). In the present study, total and progressive motility were higher in all probiotic‐supplemented groups, particularly BL and LR, suggesting beneficial effects of probiotics on sperm functional parameters. The antibiotic‐free control group exhibited lower motility, underscoring the necessity of antimicrobial components in extenders. The observed enhancement in motility and velocity parameters (VAP, VCL, VSL) suggests that probiotics, particularly BL, may exert antimicrobial and antioxidative effects, improving sperm performance. Conversely, no differences were observed among groups for LIN, STR, WOB, ALH, and BCF, indicating that probiotic inclusion did not alter sperm trajectory patterns. Progressive motility, which reflects the forward movement of spermatozoa toward the oocyte during fertilisation (Sönmez and Firat 2022). Studies in rats have reported a numerical decrease in total and progressive motility following oral probiotic supplementation (Sanchez‐Rodriguez et al. 2024), whereas human studies showed improvement in sperm motility and quality (Helli et al. 2022). Similarly, equine trials revealed no statistically significant enhancement in motility parameters (Cooke et al. 2024). In the current study, all probiotic groups exhibited higher progressive motility compared with controls. CASA analysis revealed a greater proportion of rapidly moving spermatozoa in probiotic‐treated samples than in controls, demonstrating that probiotics enhanced both motility and velocity. The proportion of static spermatozoa was highest in the control groups, while probiotic supplementation markedly reduced this proportion. Kinematic parameters such as VAP, VCL, and VSL quantify sperm velocity and movement efficiency and these measures are positively correlated (Farrell et al. 1998). The lowest values for these parameters were observed in the control groups, whereas supplementation with probiotics increased velocity, supporting the motility data. Collectively, these findings indicate that probiotic inclusion in semen diluents improves motility performance without altering the intrinsic movement characteristics of spermatozoa.

Sperm DNA integrity is a vital determinant of sperm fertilising potential, and its disruption often reveals a decline in motility and overall sperm function (Akarsu et al. 2024). Prolonged cryostress leads to excessive production of ROS, which can impair mitochondrial activity, reduce motility, and induce DNA fragmentation (Aitken and De Iuliis 2009; Fernández et al. 2003). Previous research indicates that probiotic supplementation has ameliorative effects on oxidative and genotoxic stress. As evidence of these findings, it has been reported that probiotic administration reduced sperm DNA fragmentation levels in rats exposed to a high‐fat diet (Chen et al. 2013) and improved motility and chromatin integrity in men with asthenozoospermia (Valcarce et al. 2017). In the present study, sperm DNA fragmentation exhibited a complex, strain‐dependent relationship with motility. While *Lactobacillus rhamnosus* supplementation maintained both high sperm motility and low DNA fragmentation, unexpectedly, *Bifidobacterium longum* and *Lactobacillus acidophilus* groups showed the highest levels of DNA fragmentation despite improving motility compared to controls. This apparent discrepancy is crucial for interpreting probiotic efficacy. The significantly low MDA levels and high antioxidant enzyme activities observed in the BL group confirm its strong lipid peroxidation‐mitigating capacity. However, the nuclear DNA, lacking the robust antioxidant defences present in the cytoplasm and seminal plasma, might still be vulnerable to ROS spikes that occur during the initial freezing and thawing phases. It has been suggested that the enhanced metabolic activity stimulated by certain probiotic strains, especially those displaying high motility, could transiently increase intra‐sperm ROS generation that preferentially damages the nucleus before the extrinsic antioxidant system is fully activated or can scavenge all radicals. Therefore, the high DNA fragmentation in BL, despite reduced lipid peroxidation, suggests a differential protective mechanism: BL's robust antioxidant system primarily protects the vulnerable sperm membranes but is less effective in preventing genotoxic stress to the tightly packaged chromatin. Conversely, LR appears to offer a more balanced protection across both kinetic and genomic stability, supporting the concept that motility improvement does not necessarily correlate with genomic protection and that this relationship is highly strain dependent.

Acrosomal integrity is a fundamental determinant of sperm fertilising ability, since disruption of the acrosome compromises the release of hydrolytic enzymes required for penetration of the oocyte membrane during fertilisation (Silva and Gadella 2006). In the present study, the lowest degree of acrosomal damage was recorded in *Lactobacillus acidophilus* and in the control groups without antibiotics, whereas spermatozoa from the *Lactobacillus rhamnosus* group exhibited the highest level of acrosomal defect. Although semen samples supplemented with *Bifidobacterium longum* and *Lactobacillus rhamnosus* showed superior total and progressive motility in CASA, this improvement in motility was not accompanied by preservation of acrosomal integrity. This discrepancy suggests that enhanced motility does not necessarily reflect structural stability while oxidative and osmotic stress during the freezing and thawing process may damage the acrosomal membrane displaying strong kinematic performance. The protective effect observed in the *Lactobacillus acidophilus* group may result from the membrane‐stabilising and antioxidative capacities of this probiotic strain, which can reduce lipid peroxidation and maintain osmotic balance in the sperm extender during cryopreservation. Similar findings were reported by Tvrdá et al. (2018), who demonstrated that antioxidant additives preserved acrosomal and plasma membrane integrity in cryopreserved bovine semen and by Sun et al. (2020), who showed that oxidative injury during freezing impairs acrosomal enzymes and motility in several livestock species. Furthermore, Miao et al. (2024) found that Lactobacillus‐based formulations improved post‐thaw motility and acrosomal morphology in buffalo semen, highlighting the protective potential of probiotic‐derived metabolites. Taken together, these findings suggest that *Lactobacillus acidophilus* provides dual protection by stabilising sperm membranes and maintaining acrosomal functionality, thereby ensuring both kinematic efficiency and fertilising potential during bovine semen cryopreservation.

Mitochondrial membrane potential is a key determinant of sperm function, as mitochondria located in the midpiece generate the ATP required for flagellar movement and survival; even modest depolarisation of this potential is associated with reduced motility, impaired fertilising ability and activation of apoptotic pathways. Contemporary evidence indicates that cryopreservation can disrupt mitochondrial function through oxidative stress and membrane phase transitions, leading to loss of membrane potential and decreased sperm quality in several mammalian species, including bovine. This has been clearly shown in studies linking reduced mitochondrial membrane potential with poorer motility and increased oxidative damage in spermatozoa (Amaral et al. 2013; Peña, O’Flaherty, et al. 2019). In our study, the lowest mitochondrial membrane damage was unexpectedly observed in the antibiotic‐free control group, with no significant differences among the probiotic‐supplemented groups. This finding contrasts with the significant anti‐apoptotic (lowest Caspase‐3) and anti‐oxidative (lowest MDA) effects recorded for the BL group. This apparent resilience of the MMP in the control group suggests that the basic Tris‐egg yolk extender formulation and the standard cooling protocol provided an inherent, substantial level of protection against severe membrane depolarisation, potentially minimising the measurable impact of additional components. Furthermore, the lack of significant difference in the ATP synthase enzyme among all groups further supports the idea of relative stability in the main energy‐generating machinery. We hypothesize that the powerful protective effect of BL may operate further downstream: BL's action is primarily focused on strengthening the overall redox environment (high SOD/CAT) and inhibiting the final executioners of cell death (lowering Caspase‐3), rather than significantly altering the initial mitochondrial depolarisation rate. This suggests that the observed reduction in apoptosis (Caspase‐3) in the BL group occurs independently of a dramatic change in MMP, focusing instead on mitigating cryo‐induced stress signals that lead to programmed cell death.

The integrity of the sperm plasma membrane is essential for maintaining osmotic balance, motility, and fertilisation capacity. The hypo‐osmotic swelling (HOST) test provides a sensitive indicator of functional membrane integrity by assessing the spermatozoon's ability to regulate water influx under hypo‐osmotic conditions (Přinosilová et al. 2014). Damage to the plasma membrane during freezing and thawing disrupts ion gradients and leads to loss of sperm viability and motility (Al‐Mutary 2021). In this study, the *Lactobacillus rhamnosus* groups exhibited the highest HOST response, indicating superior preservation of functional membrane integrity. This result, however, must be contextualised with LR also displaying the highest MDA and acrosome damage. The HOST specifically assesses the functional capacity of the membrane to regulate water influx (elasticity/permeability), while MDA reflects structural damage via lipid peroxidation. The co‐occurrence of high functional integrity and high biochemical damage in the LR group suggests a differential effect: LR may enhance the fluidity and elastic properties of the plasma membrane, thereby maximising its response to osmotic stress. Nevertheless, this enhanced fluidity, often associated with a higher degree of unsaturation, might simultaneously render the membrane lipids more susceptible to peroxidation by ROS during cryostress. In contrast, the lowest HOST response in the antibiotic‐free control confirms that antimicrobial components are necessary, supporting the conclusion that probiotic supplementation, particularly LR, helped maintain the plasma membrane's functional regulatory capacity.

ROS are natural by‐products of aerobic metabolism in spermatozoa and play a dual role in regulating sperm function. At physiological levels, ROS participate in essential processes such as capacitation, hyperactivation, and the acrosome reaction. However, excessive ROS generation leads to oxidative stress, resulting in lipid peroxidation, DNA fragmentation, and compromised sperm motility (Guthrie and Welch 2012). Seminal plasma contains enzymatic antioxidants such as SOD and CAT, which act synergistically to neutralise free radicals and maintain redox homeostasis. MDA, a stable by‐product of lipid peroxidation, serves as a sensitive indicator of oxidative damage in sperm membranes and testicular tissue (Gur et al. 2023). In the present study, the highest MDA levels were detected in semen samples supplemented with *Lactobacillus rhamnosus* , suggesting that this strain may have triggered pro‐oxidative effects under cryogenic stress. Conversely, the lowest MDA concentrations were observed in the *Bifidobacterium longum* group, indicating a strong antioxidative potential that effectively reduced lipid peroxidation. In agreement, SOD and CAT activities were significantly elevated in the *Bifidobacterium longum* and control groups, suggesting that this probiotic strain enhanced endogenous antioxidant defences and mitigated oxidative injury during freezing and thawing. These results align with earlier studies reporting that probiotic or antioxidant supplementation in semen extenders reduces ROS accumulation and improves post‐thaw sperm motility and viability (Al‐Mutary 2021; Tvrdá et al. 2018). Collectively, these findings imply that *Bifidobacterium longum* exerts a protective effect by strengthening the enzymatic antioxidant system and limiting oxidative membrane damage, whereas *Lactobacillus rhamnosus* may exacerbate ROS generation under cryostress, highlighting strain‐dependent differences in redox modulation during bovine sperm cryopreservation.

Spermatozoa meet their energy needs by consuming ATP (John et al. 2007). The ATP they consume is generated by mitochondria located in the tail of the spermatozoon (Sengupta et al. 2020). Damage to the mitochondria prevents the movement of spermatozoa by restricting energy formation. In particular, it has been shown that excessive levels of oxidative stress can lead to increased ATP consumption in sperm cells (Ayaz et al. 2018). In the present study, mitochondrial membrane integrity was best preserved in the control group without antibiotics, whereas no statistically significant differences were observed among the probiotic‐supplemented groups. This finding suggests that the baseline extender composition and controlled freezing conditions provided adequate protection against severe mitochondrial depolarisation, while the addition of probiotics did not further enhance mitochondrial resilience under the experimental conditions. However, considering that mitochondrial activity is closely related to ROS balance and ATP turnover, it is plausible that the observed strain‐dependent effects on oxidative stress in the probiotic groups indirectly influenced mitochondrial performance. These findings align with previous research showing that oxidative stress directly compromises mitochondrial membrane potential and ATP production in bovine spermatozoa (Amaral et al. 2013). Therefore, maintaining mitochondrial function through improved antioxidant balance remains a key target for optimising cryopreserved semen quality.

Transforming growth factor‐beta (TGF‐β) is an inflammatory inducing factor produced in the bovine vesicula seminalis. It is an important cytokine involved in immune response formation (Odhiambo et al. 2009). It has been reported that some antibiotic species cause an increase in TGF‐β levels in various tissues (Beshay et al. 2020; Wallwork et al. 2004). One study reported that the TGF‐β signalling pathway suppresses inflammation through *L. acidophilus* (Huang et al. 2015). Probiotics are also associated with a shorter inflammatory phase by accelerating the reduction of TGF‐β (Tagliari et al. 2022). These findings suggest that *Bifidobacterium longum* may attenuate cryo‐induced inflammation by modulating cytokine production and maintaining epithelial immune balance, consistent with previous reports that certain probiotic strains downregulate TGF‐β signalling and suppress pro‐inflammatory cytokine release in reproductive tissues (Feng and Wang 2020; Hekmatimoghaddam et al. 2024).

TRPM is one of the 7 subfamilies of this receptor potential. TRPM3 Bovine is responsible for reducing extracellular osmolarity (Li et al. 2010). It has been reported that sperm cryopreservation may cause changes in TRPM3 ion channels (Güngör et al. 2024). In this study, TRPM3 levels were preserved in the *Bifidobacterium longum* and control groups but significantly reduced in the *Lactobacillus rhamnosus* group, indicating that excessive oxidative stress may impair ion channel function and disrupt sperm homeostasis. Maintenance of TRPM3 integrity in the *Bifidobacterium longum* group implies enhanced membrane stability and osmotic tolerance during freezing and thawing.

The steroidogenic acute regulatory (STAR) protein is responsible for transferring cholesterol into the inner mitochondrial membrane, representing the rate‐limiting step in steroid hormone biosynthesis (Stocco 2001). Because cryopreservation induces oxidative and thermal stress, it can impair mitochondrial function and cholesterol trafficking, leading to altered steroidogenic signalling. However, in the present study, no significant differences in STAR levels were observed among groups, suggesting that the freezing–thawing process primarily affected sperm structural integrity rather than steroidogenic gene regulation. This finding aligns with previous studies reporting that spermatozoa and Leydig cells subjected to cryoinjury exhibit stable STAR expression unless exposed to prolonged oxidative stress or hormonal disruption (Park and Pang 2021). The absence of change in STAR may therefore reflect a limited or transient effect of cryostress on mitochondrial cholesterol transfer, consistent with reports that short‐term freezing procedures alter mitochondrial membrane potential without significantly modulating steroidogenic pathways (Peña, O’Flaherty, et al. 2019). These results indicate that while cryopreservation can impair mitochondrial energetics, the steroidogenic machinery remains relatively resilient under moderate oxidative conditions.

Apoptosis is programmed cell death and occurs in all tissues, including the testis (Celik et al. 2020). Caspase‐3, which plays a role in apoptosis, is a member of the cysteine aspartic acid protease family (Kankılıç et al. 2024). One study reported that sperm cryopreservation leads to apoptotic changes (Trzcińska et al. 2011). It has also been reported that the type and dose of antibiotics used in sperm cryopreservation play a role in apoptosis (Bryła and Trzcińska 2015). Moreover, another study reported that bacterial load in sperm induces apoptosis (Althouse et al. 2000). In the present study, apoptosis statistically decreased in the BL group and increased in the LR group. This is thought to occur especially after the reduction of oxidative stress by BL group bacteria.

## 5 Conclusion

In conclusion, our findings unequivocally demonstrate that *Bifidobacterium longum* (BL) represents a promising and potent antibiotic alternative for bull semen cryopreservation. This conclusion is strongly supported by BL's significant enhancement of post‐thaw motility and its superior capacity to mitigate key cryoinjury mechanisms, specifically through the profound inhibition of lipid peroxidation (MDA) and anti‐apoptotic signalling (Caspase‐3). However, the simultaneous observation of the highest DNA fragmentation in the BL group suggests a differential mode of protection, indicating that its robust antioxidant and metabolic benefits preferentially safeguard the sperm membrane and cytoplasm over the nuclear genome. This dual effect underscores a critical need for future research to elucidate strain‐specific mechanisms that ensure complete genomic stability while leveraging the established advantages of probiotic supplementation in reproductive biotechnology.

## 6 Limitations of the Study

Despite the promising in vitro results regarding probiotic supplementation in bovine semen cryopreservation, certain limitations must be acknowledged. The relatively small sample size of evaluated bulls and ejaculates necessitates larger‐scale studies to validate these findings across broader populations. Additionally, because the study primarily focused on sperm kinematics and biochemistry, sequential bacterial counts were not performed after the freeze‐thawing process, leaving the exact post‐thaw viability of the supplemented probiotic strains undetermined. The absence of in vivo fertility trials, such as non‐return rates or embryonic development tracking, further limits the ability to definitively predict the reproductive success and biosecurity of these extenders in field applications. Lastly, performing biochemical assays on the whole homogenised semen‐extender mixture means that extender components, particularly egg yolk, contributed to the baseline antioxidant readouts, thereby reflecting the total redox milieu rather than isolated intracellular sperm biochemistry.

## Author Contributions

**Ali Doğan Ömür:** conceptualisation, methodology, project administration, writing, original draft. **Demet Çelebi:** data curation. **Betül Apaydin Yildirim:** formal analysis. **Gamze Uçak:** investigation, data curation, writing, original draft. **Tutku Can Acisu:** methodology. **Mehmet Akif Aydin:** resources. **Sümeyye Başer:** investigation. **Özgür Çelebi:** formal analysis. **Recep Hakkı Koca:** data curation. **Gözde Arkali:** formal analysis. **Serkan Ali Akarsu:** conceptualisation, resources, writing, original draft, editing. **Tarique Hussain:** review and editing. All co‐authors have reviewed and approved the manuscript before submission.

## Funding

This work was supported by Türkiye Bilimsel ve Teknolojik Araştırma Kurumu (Grant 123O636).

## Conflicts of Interest

The authors declare no conflicts of interest.

## Data Availability

The data used to support the findings of this study are available from the corresponding author upon reasonable request.
